# Tetra­kis(μ-2-chloro-4-nitro­benzoato-κ^2^ *O*:*O*′)bis­[aqua­copper(II)]

**DOI:** 10.1107/S1600536808041986

**Published:** 2008-12-17

**Authors:** Eng Khoon Lim, Siang Guan Teoh, Ibrahim Abdul Razak, Samuel Robinson Jebas, Hoong-Kun Fun

**Affiliations:** a School of Chemical Sciences, Universiti Sains Malaysia, 11800 USM, Penang, Malaysia; b X-ray Crystallography Unit, School of Physics, Universiti Sains Malaysia, 11800 USM, Penang, Malaysia

## Abstract

In the title binuclear copper(II) complex, [Cu_2_(C_7_H_3_ClNO_4_)_4_(H_2_O)_2_], each of the two independent Cu^II^ center is five-coordinated by four O atoms of the carboxyl­ate groups in the basal plane and one O atom of a water mol­ecule in the apical position, in a distorted square-pyramidal geometry. The Cu—Cu distance is 2.6458 (4) Å. In the crystal structure, the dinuclear units are linked into a three-dimensional network by O—H⋯O, C—H⋯O and C—H⋯Cl hydrogen bonds. One of the Cl atoms is disordered over two positions with occupancies of 0.650 (2) and 0.350 (2).

## Related literature

For general background, see: Balaraman *et al.* (2006 ▶). For bond-length data, see: Allen *et al.* (1987 ▶). For related structures, see: Kabbani *et al.* (2004 ▶); Stachová *et al.* (2004 ▶).
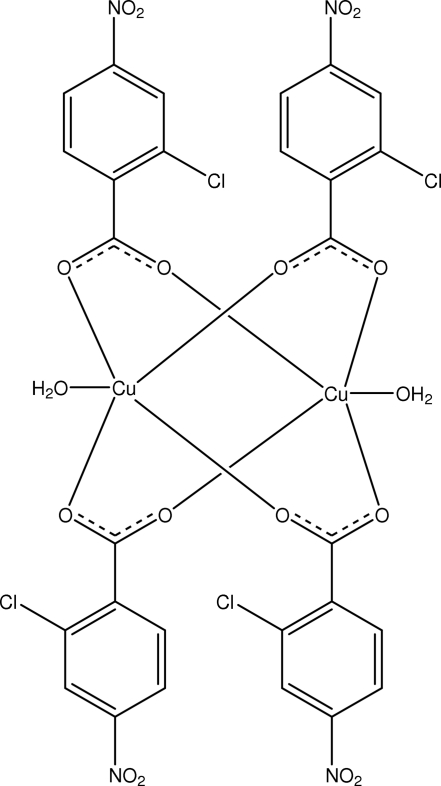


## Experimental

### 

#### Crystal data

[Cu_2_(C_7_H_3_ClNO_4_)_4_(H_2_O)_2_]
*M* *_r_* = 965.33
Monoclinic, 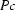

*a* = 7.6721 (2) Å
*b* = 15.2938 (4) Å
*c* = 14.5653 (3) Å
β = 102.327 (1)°
*V* = 1669.63 (7) Å^3^
*Z* = 2
Mo *K*α radiation
μ = 1.69 mm^−1^
*T* = 293 (2) K
0.76 × 0.19 × 0.10 mm

#### Data collection

Bruker SMART APEXII CCD area-detector diffractometer
Absorption correction: multi-scan (*SADABS*; Bruker, 2005 ▶) *T*_min_ = 0.360, *T*_max_ = 0.844
61401 measured reflections
18745 independent reflections
13379 reflections with *I* > 2σ(*I*)
*R*_int_ = 0.041

#### Refinement

*R*[*F* ^2^ > 2σ(*F* ^2^)] = 0.049
*wR*(*F* ^2^) = 0.121
*S* = 1.11
18745 reflections
516 parameters
2 restraints
H-atom parameters constrained
Δρ_max_ = 1.75 e Å^−3^
Δρ_min_ = −0.85 e Å^−3^
Absolute structure: Flack (1983 ▶), 8242 Friedel pairs
Flack parameter: 0.526 (8)

### 

Data collection: *APEX2* (Bruker, 2005 ▶); cell refinement: *SAINT* (Bruker, 2005 ▶); data reduction: *SAINT*; program(s) used to solve structure: *SHELXTL* (Sheldrick, 2008 ▶); program(s) used to refine structure: *SHELXTL*; molecular graphics: *SHELXTL*; software used to prepare material for publication: *SHELXTL* and *PLATON* (Spek, 2003 ▶).

## Supplementary Material

Crystal structure: contains datablocks global, I. DOI: 10.1107/S1600536808041986/ci2732sup1.cif

Structure factors: contains datablocks I. DOI: 10.1107/S1600536808041986/ci2732Isup2.hkl

Additional supplementary materials:  crystallographic information; 3D view; checkCIF report

## Figures and Tables

**Table 1:** Selected bond lengths (Å)

Cu1—O7	1.953 (2)
Cu1—O4	1.967 (2)
Cu1—O5	1.971 (2)
Cu1—O2	1.983 (2)
Cu1—O2*W*	2.159 (2)
Cu2—O3	1.948 (2)
Cu2—O8	1.964 (2)
Cu2—O1	1.968 (2)
Cu2—O6	1.987 (2)
Cu2—O1*W*	2.137 (2)

**Table 2:** Hydrogen-bond geometry (Å, °)

*D*—H⋯*A*	*D*—H	H⋯*A*	*D*⋯*A*	*D*—H⋯*A*
O1*W*—H1*W*1⋯O13^i^	0.85	2.35	2.910 (3)	124
O1*W*—H2*W*1⋯O2^ii^	0.85	1.99	2.838 (3)	175
O2*W*—H1*W*2⋯O9^iii^	0.82	2.15	2.927 (3)	158
O2*W*—H2*W*2⋯O6^iv^	0.85	1.98	2.826 (3)	173
C1—H1*A*⋯Cl1^v^	0.93	2.78	3.417 (3)	127
C4—H4*A*⋯O14^vi^	0.93	2.51	3.331 (4)	147
C8—H8*A*⋯O12^v^	0.93	2.38	3.269 (4)	159
C18—H18*A*⋯O10^vii^	0.93	2.55	3.364 (4)	147
C22—H22*A*⋯O16^viii^	0.93	2.36	3.240 (4)	158
C23—H23*A*⋯O2*W*^i^	0.93	2.51	3.385 (3)	157

Symmetry codes: (i) 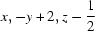; (ii) 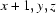; (iii) 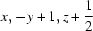; (iv) 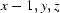; (v) 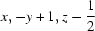; (vi) 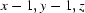; (vii) 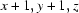; (viii) 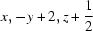.

**Fig. 1.**
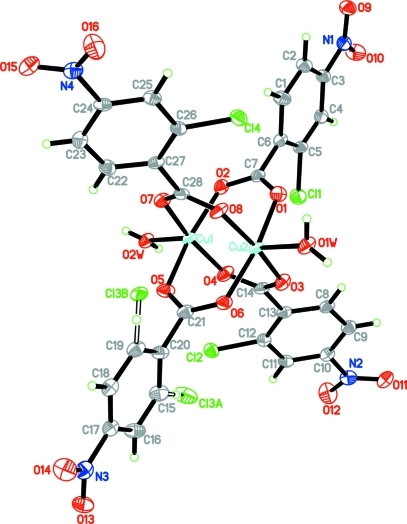
The molecular structure of the title compound, showing 50% probability displacement ellipsoids and the atom-numbering scheme. Both disorder components are given.

**Fig. 2.**
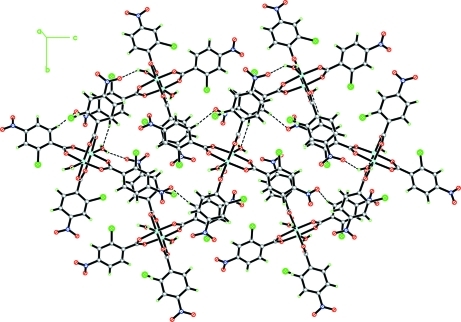
The crystal packing of the title compound, viewed down the a axis. Hydrogen bonds are shown as dotted lines. Only the major disorder component is shown.

## supplementary crystallographic information

### Comment

In our quest to study the biological properties of Cu^II^ complexes, we have managed to prepare several water soluble Cu^II^ complexes. Cu^II^ complexes have been known to exhibit DNA cleavage activity *in vitro* (Balaraman *et al.*, 2006). Herein, we report the preparation and crystal structure of the title compound.

The coordination geometry around each Cu^II^ atom is square-pyramidal. Four O atoms, one each from the carboxylate groups of four organic ligands, form the basal plane with an average Cu—O bond distance of 1.968 (2) Å. The O atom of the water molecules lie in the apical position with an average Cu—O distance of 2.148 (2) Å. The Cu1 and Cu2 atoms are slightly displaced from the corresponding basal plane by 0.2037 (2) and 0.1959 (2) Å, respectively. The Cu1—Cu2 distance is 2.6458 (4) Å. Similar characteristics of the copper atom were also reported by Kabbani *et al.* (2004) and Stachová *et al.* (2004).

Bond lengths in the ligand show normal values (Allen *et al.*, 1987). Dihedral angles between nitro groups and benzene rings are: 6.3 (4) [C1-C6/O9,O10,N1], 16.0 (3) [C8-C13/O11,O12,N2], 6.5 (4) [C15-C20/O13,O14,N3] and 19.9 (3) Å [C22-C27/O15,O16,N4].

In the crystal structure, O—H···O, C—H···O and C—H···Cl intermolecular interactions (Table 2) form a three-dimensional network (Fig.2).

### Experimental

An ethanol solution (50 ml) of 2-chloro-4-nitrobenzoic acid (4.84 g, 0.024 mol) was added to a solution of copper(II) sulfate pentahydrate (3.00 g, 0.012 mol) in ethanol (50 ml). This mixture was then stirred and refluxed and left to cool down to room temperature. After a few days of slow evaporation, blue crystals which are suitable for X-ray analysis were collected.

### Refinement

Water H atoms were located in a difference Fourier map and were allowed to ride on the O atom, with U_iso_(H) = 1.5U_eq_(O). All other H atoms were positioned geometrically and refined using a riding model, with C-H = 0.93 Å and *U*_iso_(H) = 1.5*U*_eq_(C). Atom Cl3 attached to one of the phenyl rings is disordered over two positions with occupancies of 0.650 (2) and 0.350 (2). The structure shows a pseudo centre of symmetry. It can be solved and refined in the space group P2_1_/c but the final *R* value (0.098) is large. The highest residual electron density peak is located at 1.02 Å from Cl3A and the deepest hole is located at 0.63Å from Cu1. The crystal is a twin with BASF = 0.526 (8).

### Crystal data

**Table:**

[Cu_2_(C_7_H_3_ClNO_4_)_4_(H_2_O)_2_]	*F*(000) = 964
*M**_r_* = 965.33	*D*_x_ = 1.920 Mg m^−^^3^
Monoclinic, *P**c*	Mo *K*α radiation, λ = 0.71073 Å
Hall symbol: P -2yc	Cell parameters from 7272 reflections
*a* = 7.6721 (2) Å	θ = 2.7–38.1°
*b* = 15.2938 (4) Å	µ = 1.69 mm^−^^1^
*c* = 14.5653 (3) Å	*T* = 293 K
β = 102.327 (1)°	Block, blue
*V* = 1669.63 (7) Å^3^	0.76 × 0.19 × 0.10 mm
*Z* = 2	

### Data collection

**Table:**

Bruker SMART APEXII CCD area-detector diffractometer	18745 independent reflections
Radiation source: fine-focus sealed tube	13379 reflections with *I* > 2σ(*I*)
graphite	*R*_int_ = 0.041
φ and ω scans	θ_max_ = 40.5°, θ_min_ = 1.3°
Absorption correction: multi-scan (*SADABS*; Bruker, 2005)	*h* = −13→13
*T*_min_ = 0.360, *T*_max_ = 0.844	*k* = −27→27
61401 measured reflections	*l* = −26→26

### Refinement

**Table:**

Refinement on *F*^2^	Secondary atom site location: difference Fourier map
Least-squares matrix: full	Hydrogen site location: inferred from neighbouring sites
*R*[*F*^2^ > 2σ(*F*^2^)] = 0.049	H-atom parameters constrained
*wR*(*F*^2^) = 0.121	*w* = 1/[σ^2^(*F*_o_^2^) + (0.0545*P*)^2^] where *P* = (*F*_o_^2^ + 2*F*_c_^2^)/3
*S* = 1.11	(Δ/σ)_max_ = 0.001
18745 reflections	Δρ_max_ = 1.75 e Å^−^^3^
516 parameters	Δρ_min_ = −0.85 e Å^−^^3^
2 restraints	Absolute structure: Flack (1983), 8242 Friedel pairs
Primary atom site location: structure-invariant direct methods	Flack parameter: 0.526 (8)

### Special details

**Table:**

Geometry. All e.s.d.'s (except the e.s.d. in the dihedral angle between two l.s. planes) are estimated using the full covariance matrix. The cell e.s.d.'s are taken into account individually in the estimation of e.s.d.'s in distances, angles and torsion angles; correlations between e.s.d.'s in cell parameters are only used when they are defined by crystal symmetry. An approximate (isotropic) treatment of cell e.s.d.'s is used for estimating e.s.d.'s involving l.s. planes.
Refinement. Refinement of *F*^2^ against ALL reflections. The weighted *R*-factor *wR* and goodness of fit *S* are based on *F*^2^, conventional *R*-factors *R* are based on *F*, with *F* set to zero for negative *F*^2^. The threshold expression of *F*^2^ > σ(*F*^2^) is used only for calculating *R*-factors(gt) *etc*. and is not relevant to the choice of reflections for refinement. *R*-factors based on *F*^2^ are statistically about twice as large as those based on *F*, and *R*- factors based on ALL data will be even larger.

### Fractional atomic coordinates and isotropic or equivalent isotropic displacement parameters (Å^2^)

**Table:**

	*x*	*y*	*z*	*U*_iso_*/*U*_eq_	Occ. (<1)
Cu1	0.12722 (3)	0.783035 (19)	0.823603 (19)	0.01681 (6)	
Cu2	0.43478 (3)	0.72693 (2)	0.791272 (19)	0.01749 (6)	
Cl1	0.09737 (12)	0.49165 (5)	0.90348 (5)	0.02859 (15)	
Cl2	0.26779 (10)	0.78853 (4)	1.14338 (5)	0.02285 (13)	
Cl3A	0.51796 (19)	0.94108 (7)	1.07198 (7)	0.0375 (3)	0.650 (2)
H19A	0.4968	1.0306	0.7621	0.045*	0.650 (2)
Cl3B	0.4619 (3)	1.00985 (13)	0.71300 (12)	0.0219 (4)	0.350 (2)
H15A	0.5376	0.9693	1.0367	0.026*	0.350 (2)
Cl4	0.28627 (11)	0.71075 (4)	0.47526 (5)	0.02455 (14)	
O1	0.2962 (3)	0.62123 (13)	0.74654 (14)	0.0232 (4)	
O2	0.0341 (3)	0.66738 (13)	0.77463 (14)	0.0208 (4)	
O3	0.4781 (3)	0.67950 (13)	0.91825 (14)	0.0231 (4)	
O4	0.2187 (3)	0.72835 (14)	0.94666 (15)	0.0242 (4)	
O5	0.2647 (3)	0.89022 (14)	0.86431 (15)	0.0245 (4)	
O6	0.5291 (3)	0.84210 (12)	0.84230 (14)	0.0216 (4)	
O7	0.0803 (3)	0.82590 (13)	0.69431 (13)	0.0205 (4)	
O8	0.3478 (3)	0.78407 (13)	0.66932 (15)	0.0234 (4)	
O9	−0.1850 (3)	0.25394 (14)	0.55559 (15)	0.0271 (4)	
O10	−0.1868 (4)	0.22569 (14)	0.70107 (18)	0.0326 (5)	
O11	0.6789 (3)	0.47095 (14)	1.34252 (16)	0.0284 (4)	
O12	0.5010 (4)	0.55861 (16)	1.39558 (16)	0.0381 (6)	
O13	0.7524 (3)	1.25825 (14)	1.05715 (16)	0.0295 (4)	
O14	0.7563 (4)	1.28442 (14)	0.91156 (19)	0.0354 (6)	
O15	−0.1241 (3)	1.02192 (14)	0.26224 (15)	0.0282 (4)	
O16	0.0681 (4)	0.93987 (16)	0.21492 (17)	0.0414 (7)	
N1	−0.1544 (4)	0.27333 (14)	0.63933 (19)	0.0218 (5)	
N2	0.5679 (3)	0.52972 (16)	1.33285 (17)	0.0239 (5)	
N3	0.7216 (4)	1.23743 (17)	0.9736 (2)	0.0250 (5)	
N4	−0.0059 (3)	0.96664 (15)	0.27622 (17)	0.0224 (5)	
C1	0.0091 (4)	0.50152 (19)	0.6236 (2)	0.0243 (5)	
H1A	0.0222	0.5424	0.5783	0.029*	
C2	−0.0583 (4)	0.41837 (18)	0.59602 (19)	0.0218 (5)	
H2A	−0.0893	0.4028	0.5329	0.026*	
C3	−0.0775 (4)	0.35938 (17)	0.66677 (19)	0.0198 (5)	
C4	−0.0312 (4)	0.38101 (17)	0.76208 (19)	0.0204 (5)	
H4A	−0.0456	0.3409	0.8078	0.024*	
C5	0.0357 (4)	0.46238 (18)	0.78645 (18)	0.0197 (5)	
C6	0.0573 (3)	0.52397 (17)	0.71939 (18)	0.0178 (4)	
C7	0.1335 (3)	0.61089 (17)	0.74809 (18)	0.0182 (4)	
C8	0.4969 (4)	0.56868 (18)	1.0754 (2)	0.0223 (5)	
H8A	0.5226	0.5419	1.0225	0.027*	
C9	0.5457 (4)	0.52623 (19)	1.1623 (2)	0.0235 (5)	
H9A	0.6004	0.4717	1.1675	0.028*	
C10	0.5096 (4)	0.56815 (17)	1.23927 (19)	0.0197 (5)	
C11	0.4237 (4)	0.64803 (17)	1.23367 (19)	0.0199 (5)	
H11A	0.4004	0.6746	1.2872	0.024*	
C12	0.3726 (4)	0.68801 (17)	1.14587 (19)	0.0184 (5)	
C13	0.4121 (3)	0.64894 (18)	1.06542 (19)	0.0193 (5)	
C14	0.3656 (3)	0.69000 (18)	0.97047 (18)	0.0194 (5)	
C15	0.5526 (4)	1.0118 (2)	0.9896 (2)	0.0254 (6)	
C16	0.6210 (4)	1.0937 (2)	1.0165 (2)	0.0267 (6)	
H16A	0.6517	1.1095	1.0795	0.032*	
C17	0.6422 (4)	1.15070 (18)	0.94707 (19)	0.0208 (5)	
C18	0.5973 (4)	1.12999 (18)	0.8526 (2)	0.0222 (5)	
H18A	0.6125	1.1703	0.8072	0.027*	
C19	0.5287 (4)	1.04701 (19)	0.8275 (2)	0.0239 (5)	
C20	0.5060 (4)	0.98790 (18)	0.8952 (2)	0.0217 (5)	
C21	0.4268 (4)	0.89879 (19)	0.86537 (19)	0.0205 (5)	
C22	0.0576 (4)	0.93515 (17)	0.5342 (2)	0.0224 (5)	
H22A	0.0303	0.9632	0.5860	0.027*	
C23	0.0106 (4)	0.97402 (17)	0.4471 (2)	0.0208 (5)	
H23A	−0.0451	1.0283	0.4394	0.025*	
C24	0.0497 (4)	0.92867 (17)	0.37110 (18)	0.0190 (5)	
C25	0.1356 (4)	0.84924 (16)	0.37889 (19)	0.0197 (5)	
H25A	0.1600	0.8210	0.3265	0.024*	
C26	0.1848 (4)	0.81247 (17)	0.46865 (18)	0.0183 (5)	
C27	0.1456 (3)	0.85452 (17)	0.54673 (18)	0.0172 (4)	
C28	0.1957 (4)	0.81801 (16)	0.64518 (18)	0.0180 (4)	
O1W	0.6588 (3)	0.69210 (14)	0.73336 (14)	0.0239 (4)	
H1W1	0.6256	0.6795	0.6754	0.036*	
H2W1	0.7716	0.6869	0.7482	0.036*	
O2W	−0.0975 (3)	0.81661 (13)	0.88410 (14)	0.0207 (4)	
H1W2	−0.1240	0.7839	0.9236	0.031*	
H2W2	−0.2089	0.8273	0.8676	0.031*	

### Atomic displacement parameters (Å^2^)

**Table:**

	*U*^11^	*U*^22^	*U*^33^	*U*^12^	*U*^13^	*U*^23^
Cu1	0.01168 (13)	0.02709 (15)	0.01187 (14)	0.00096 (10)	0.00296 (10)	−0.00209 (11)
Cu2	0.01168 (13)	0.02907 (15)	0.01193 (14)	0.00102 (10)	0.00297 (10)	−0.00302 (11)
Cl1	0.0377 (4)	0.0294 (3)	0.0179 (3)	0.0038 (3)	0.0042 (3)	0.0002 (3)
Cl2	0.0288 (4)	0.0221 (3)	0.0180 (3)	0.0003 (2)	0.0059 (3)	−0.0009 (2)
Cl3A	0.0666 (8)	0.0300 (5)	0.0146 (4)	−0.0177 (5)	0.0059 (4)	0.0024 (4)
Cl3B	0.0266 (10)	0.0296 (10)	0.0077 (7)	0.0037 (7)	−0.0005 (6)	−0.0008 (6)
Cl4	0.0340 (4)	0.0204 (3)	0.0189 (3)	0.0019 (2)	0.0048 (3)	−0.0025 (2)
O1	0.0162 (9)	0.0313 (10)	0.0243 (10)	−0.0004 (7)	0.0089 (7)	−0.0065 (7)
O2	0.0155 (8)	0.0259 (9)	0.0214 (10)	0.0022 (6)	0.0050 (7)	−0.0006 (7)
O3	0.0174 (10)	0.0350 (11)	0.0181 (10)	0.0051 (7)	0.0061 (7)	0.0018 (8)
O4	0.0164 (10)	0.0409 (12)	0.0156 (10)	0.0049 (7)	0.0037 (8)	0.0009 (7)
O5	0.0152 (8)	0.0335 (10)	0.0250 (11)	−0.0023 (7)	0.0049 (7)	−0.0111 (8)
O6	0.0140 (8)	0.0286 (9)	0.0221 (10)	−0.0005 (6)	0.0033 (7)	−0.0064 (7)
O7	0.0197 (10)	0.0294 (10)	0.0129 (9)	0.0013 (7)	0.0048 (7)	−0.0006 (7)
O8	0.0142 (9)	0.0408 (12)	0.0142 (10)	0.0030 (6)	0.0007 (7)	0.0011 (7)
O9	0.0290 (11)	0.0311 (10)	0.0216 (10)	0.0008 (8)	0.0063 (8)	−0.0050 (8)
O10	0.0419 (15)	0.0304 (11)	0.0262 (12)	−0.0101 (8)	0.0089 (11)	0.0022 (8)
O11	0.0216 (10)	0.0359 (11)	0.0266 (11)	0.0012 (7)	0.0028 (8)	0.0083 (8)
O12	0.0580 (17)	0.0405 (13)	0.0205 (11)	0.0081 (11)	0.0190 (11)	0.0083 (9)
O13	0.0337 (12)	0.0340 (11)	0.0215 (11)	−0.0052 (9)	0.0077 (8)	−0.0077 (9)
O14	0.0453 (16)	0.0361 (13)	0.0281 (13)	−0.0081 (9)	0.0153 (12)	0.0046 (9)
O15	0.0242 (11)	0.0353 (11)	0.0231 (10)	−0.0008 (8)	0.0009 (8)	0.0066 (8)
O16	0.076 (2)	0.0343 (12)	0.0192 (11)	0.0149 (12)	0.0221 (12)	0.0073 (9)
N1	0.0219 (11)	0.0263 (11)	0.0188 (11)	0.0021 (7)	0.0075 (9)	−0.0002 (8)
N2	0.0272 (13)	0.0280 (11)	0.0172 (11)	−0.0048 (8)	0.0063 (9)	0.0034 (9)
N3	0.0198 (12)	0.0307 (11)	0.0258 (13)	0.0020 (8)	0.0081 (10)	0.0002 (10)
N4	0.0266 (12)	0.0240 (11)	0.0167 (10)	−0.0054 (8)	0.0048 (9)	0.0034 (8)
C1	0.0299 (14)	0.0268 (12)	0.0173 (13)	−0.0025 (9)	0.0077 (10)	−0.0023 (9)
C2	0.0277 (13)	0.0249 (12)	0.0141 (11)	−0.0001 (9)	0.0077 (9)	−0.0012 (9)
C3	0.0203 (12)	0.0246 (12)	0.0157 (11)	0.0012 (8)	0.0063 (9)	−0.0028 (9)
C4	0.0220 (12)	0.0248 (12)	0.0155 (12)	0.0032 (9)	0.0067 (9)	0.0007 (9)
C5	0.0184 (11)	0.0288 (12)	0.0114 (11)	0.0029 (8)	0.0024 (8)	−0.0024 (9)
C6	0.0138 (10)	0.0278 (12)	0.0129 (11)	0.0028 (8)	0.0052 (8)	−0.0017 (8)
C7	0.0160 (11)	0.0258 (12)	0.0125 (11)	0.0015 (8)	0.0019 (8)	−0.0012 (8)
C8	0.0188 (12)	0.0352 (14)	0.0120 (11)	0.0036 (9)	0.0015 (8)	−0.0043 (10)
C9	0.0207 (13)	0.0299 (13)	0.0198 (13)	0.0010 (9)	0.0041 (10)	−0.0017 (10)
C10	0.0162 (11)	0.0238 (12)	0.0196 (13)	−0.0031 (8)	0.0051 (9)	0.0031 (9)
C11	0.0217 (13)	0.0252 (12)	0.0135 (11)	−0.0042 (9)	0.0056 (9)	−0.0023 (9)
C12	0.0173 (11)	0.0198 (11)	0.0188 (13)	−0.0034 (8)	0.0053 (9)	0.0005 (9)
C13	0.0140 (11)	0.0285 (12)	0.0152 (12)	−0.0031 (8)	0.0025 (9)	−0.0030 (9)
C14	0.0148 (11)	0.0297 (12)	0.0135 (11)	−0.0032 (8)	0.0029 (8)	−0.0042 (9)
C15	0.0277 (14)	0.0334 (14)	0.0161 (13)	−0.0038 (10)	0.0066 (10)	0.0000 (10)
C16	0.0289 (14)	0.0353 (14)	0.0161 (13)	−0.0077 (10)	0.0052 (10)	−0.0032 (10)
C17	0.0182 (12)	0.0283 (12)	0.0171 (12)	0.0024 (8)	0.0064 (9)	−0.0017 (9)
C18	0.0196 (12)	0.0284 (13)	0.0195 (13)	0.0020 (9)	0.0061 (10)	0.0030 (10)
C19	0.0200 (12)	0.0363 (14)	0.0156 (12)	0.0026 (10)	0.0045 (9)	−0.0028 (10)
C20	0.0173 (11)	0.0287 (13)	0.0189 (13)	−0.0017 (8)	0.0037 (9)	−0.0065 (10)
C21	0.0161 (11)	0.0325 (13)	0.0133 (11)	0.0006 (9)	0.0039 (9)	−0.0035 (9)
C22	0.0221 (13)	0.0250 (12)	0.0211 (13)	0.0002 (9)	0.0070 (10)	−0.0043 (10)
C23	0.0202 (12)	0.0219 (12)	0.0213 (13)	0.0013 (8)	0.0067 (10)	0.0014 (9)
C24	0.0212 (13)	0.0242 (12)	0.0117 (11)	−0.0062 (9)	0.0039 (9)	−0.0016 (9)
C25	0.0244 (13)	0.0215 (11)	0.0143 (12)	−0.0036 (9)	0.0063 (10)	−0.0005 (9)
C26	0.0197 (12)	0.0220 (11)	0.0142 (12)	−0.0009 (8)	0.0055 (9)	−0.0027 (8)
C27	0.0167 (12)	0.0240 (11)	0.0107 (10)	−0.0022 (8)	0.0026 (8)	−0.0008 (8)
C28	0.0183 (12)	0.0215 (11)	0.0130 (11)	−0.0019 (8)	0.0005 (9)	−0.0038 (8)
O1W	0.0138 (9)	0.0420 (11)	0.0171 (9)	0.0041 (7)	0.0060 (7)	0.0002 (8)
O2W	0.0151 (9)	0.0296 (10)	0.0184 (9)	0.0010 (6)	0.0055 (7)	0.0006 (7)

### Geometric parameters (Å, °)

**Table:**

Cu1—O7	1.953 (2)	C2—H2A	0.93
Cu1—O4	1.967 (2)	C3—C4	1.397 (4)
Cu1—O5	1.971 (2)	C4—C5	1.364 (4)
Cu1—O2	1.983 (2)	C4—H4A	0.93
Cu1—O2W	2.159 (2)	C5—C6	1.392 (4)
Cu1—Cu2	2.6458 (3)	C6—C7	1.476 (4)
Cu2—O3	1.948 (2)	C8—C13	1.382 (4)
Cu2—O8	1.964 (2)	C8—C9	1.400 (4)
Cu2—O1	1.968 (2)	C8—H8A	0.93
Cu2—O6	1.987 (2)	C9—C10	1.370 (4)
Cu2—O1W	2.137 (2)	C9—H9A	0.93
Cl1—C5	1.728 (3)	C10—C11	1.382 (4)
Cl2—C12	1.732 (3)	C11—C12	1.396 (4)
Cl3A—C15	1.678 (3)	C11—H11A	0.93
Cl3B—C19	1.733 (3)	C12—C13	1.405 (4)
Cl3B—H19A	0.7759	C13—C14	1.491 (4)
Cl4—C26	1.733 (3)	C15—C16	1.382 (4)
O1—C7	1.263 (3)	C15—C20	1.393 (4)
O2—C7	1.266 (3)	C15—H15A	0.97
O3—C14	1.277 (3)	C16—C17	1.371 (4)
O4—C14	1.252 (3)	C16—H16A	0.93
O5—C21	1.247 (3)	C17—C18	1.381 (4)
O6—C21	1.262 (3)	C18—C19	1.392 (4)
O7—C28	1.257 (3)	C18—H18A	0.93
O8—C28	1.257 (3)	C19—C20	1.376 (4)
O9—N1	1.228 (3)	C19—H19A	0.96
O10—N1	1.223 (3)	C20—C21	1.518 (4)
O11—N2	1.225 (3)	C22—C23	1.378 (4)
O12—N2	1.223 (3)	C22—C27	1.399 (4)
O13—N3	1.232 (3)	C22—H22A	0.93
O14—N3	1.227 (4)	C23—C24	1.393 (4)
O15—N4	1.224 (3)	C23—H23A	0.93
O16—N4	1.226 (3)	C24—C25	1.375 (4)
N1—C3	1.462 (4)	C25—C26	1.399 (4)
N2—C10	1.464 (4)	C25—H25A	0.93
N3—C17	1.476 (4)	C26—C27	1.394 (4)
N4—C24	1.476 (4)	C27—C28	1.510 (4)
C1—C2	1.399 (4)	O1W—H1W1	0.85
C1—C6	1.407 (4)	O1W—H2W1	0.85
C1—H1A	0.93	O2W—H1W2	0.82
C2—C3	1.401 (4)	O2W—H2W2	0.85
			
O7—Cu1—O4	167.90 (9)	C13—C8—H8A	118.8
O7—Cu1—O5	89.29 (9)	C9—C8—H8A	118.8
O4—Cu1—O5	90.90 (10)	C10—C9—C8	117.5 (3)
O7—Cu1—O2	88.53 (9)	C10—C9—H9A	121.3
O4—Cu1—O2	88.83 (9)	C8—C9—H9A	121.3
O5—Cu1—O2	168.24 (8)	C9—C10—C11	122.8 (3)
O7—Cu1—O2W	108.34 (8)	C9—C10—N2	119.9 (3)
O4—Cu1—O2W	83.68 (8)	C11—C10—N2	117.2 (2)
O5—Cu1—O2W	95.85 (8)	C10—C11—C12	118.6 (2)
O2—Cu1—O2W	95.81 (8)	C10—C11—H11A	120.7
O7—Cu1—Cu2	85.71 (6)	C12—C11—H11A	120.7
O4—Cu1—Cu2	82.30 (7)	C11—C12—C13	120.6 (2)
O5—Cu1—Cu2	83.29 (6)	C11—C12—Cl2	116.5 (2)
O2—Cu1—Cu2	85.02 (6)	C13—C12—Cl2	122.8 (2)
O2W—Cu1—Cu2	165.93 (6)	C8—C13—C12	118.1 (3)
O3—Cu2—O8	168.65 (9)	C8—C13—C14	119.0 (2)
O3—Cu2—O1	88.80 (9)	C12—C13—C14	122.9 (2)
O8—Cu2—O1	90.62 (9)	O4—C14—O3	125.2 (3)
O3—Cu2—O6	90.01 (9)	O4—C14—C13	118.5 (2)
O8—Cu2—O6	88.28 (9)	O3—C14—C13	116.2 (2)
O1—Cu2—O6	168.47 (8)	C16—C15—C20	121.2 (3)
O3—Cu2—O1W	107.06 (8)	C16—C15—Cl3A	119.3 (2)
O8—Cu2—O1W	84.29 (9)	C20—C15—Cl3A	119.4 (2)
O1—Cu2—O1W	95.03 (8)	C16—C15—H15A	120.1
O6—Cu2—O1W	96.28 (9)	C20—C15—H15A	118.6
O3—Cu2—Cu1	85.97 (6)	C17—C16—C15	117.7 (3)
O8—Cu2—Cu1	82.70 (7)	C17—C16—H16A	121.1
O1—Cu2—Cu1	83.46 (6)	C15—C16—H16A	121.1
O6—Cu2—Cu1	85.02 (6)	C16—C17—C18	123.0 (3)
O1W—Cu2—Cu1	166.88 (6)	C16—C17—N3	119.1 (3)
C7—O1—Cu2	124.26 (18)	C18—C17—N3	117.9 (3)
C7—O2—Cu1	121.62 (18)	C17—C18—C19	118.1 (3)
C14—O3—Cu2	121.26 (18)	C17—C18—H18A	121.0
C14—O4—Cu1	125.22 (19)	C19—C18—H18A	121.0
C21—O5—Cu1	124.00 (19)	C20—C19—C18	120.6 (3)
C21—O6—Cu2	120.73 (18)	C20—C19—Cl3B	114.7 (2)
C28—O7—Cu1	120.60 (18)	C18—C19—Cl3B	124.7 (2)
C28—O8—Cu2	123.54 (19)	C20—C19—H19A	119.5
O10—N1—O9	124.1 (3)	C18—C19—H19A	119.9
O10—N1—C3	117.9 (3)	C19—C20—C15	119.3 (3)
O9—N1—C3	118.0 (2)	C19—C20—C21	119.3 (2)
O12—N2—O11	124.9 (3)	C15—C20—C21	121.4 (3)
O12—N2—C10	117.2 (3)	O5—C21—O6	126.8 (3)
O11—N2—C10	117.9 (2)	O5—C21—C20	115.8 (2)
O14—N3—O13	123.4 (3)	O6—C21—C20	117.3 (2)
O14—N3—C17	118.4 (3)	C23—C22—C27	121.6 (2)
O13—N3—C17	118.2 (2)	C23—C22—H22A	119.2
O15—N4—O16	123.4 (3)	C27—C22—H22A	119.2
O15—N4—C24	118.9 (2)	C22—C23—C24	117.3 (2)
O16—N4—C24	117.7 (3)	C22—C23—H23A	121.3
C2—C1—C6	120.6 (3)	C24—C23—H23A	121.3
C2—C1—H1A	119.7	C25—C24—C23	123.7 (3)
C6—C1—H1A	119.7	C25—C24—N4	117.6 (2)
C1—C2—C3	117.7 (3)	C23—C24—N4	118.7 (2)
C1—C2—H2A	121.2	C24—C25—C26	117.5 (2)
C3—C2—H2A	121.2	C24—C25—H25A	121.3
C4—C3—C2	122.3 (3)	C26—C25—H25A	121.3
C4—C3—N1	119.1 (2)	C27—C26—C25	121.0 (2)
C2—C3—N1	118.6 (3)	C27—C26—Cl4	122.8 (2)
C5—C4—C3	118.5 (2)	C25—C26—Cl4	116.18 (19)
C5—C4—H4A	120.8	C26—C27—C22	118.9 (2)
C3—C4—H4A	120.8	C26—C27—C28	123.3 (2)
C4—C5—C6	122.0 (2)	C22—C27—C28	117.9 (2)
C4—C5—Cl1	120.1 (2)	O8—C28—O7	127.2 (3)
C6—C5—Cl1	117.9 (2)	O8—C28—C27	116.8 (2)
C5—C6—C1	119.0 (2)	O7—C28—C27	116.0 (2)
C5—C6—C7	120.7 (2)	Cu2—O1W—H1W1	110.7
C1—C6—C7	120.3 (2)	Cu2—O1W—H2W1	141.6
O1—C7—O2	125.6 (3)	H1W1—O1W—H2W1	107.7
O1—C7—C6	116.1 (2)	Cu1—O2W—H1W2	118.7
O2—C7—C6	118.2 (2)	Cu1—O2W—H2W2	140.4
C13—C8—C9	122.4 (3)	H1W2—O2W—H2W2	86.0
			
O7—Cu1—Cu2—O3	−177.67 (10)	Cu1—O2—C7—O1	−2.3 (4)
O4—Cu1—Cu2—O3	0.72 (9)	Cu1—O2—C7—C6	175.39 (18)
O5—Cu1—Cu2—O3	92.54 (9)	C5—C6—C7—O1	100.4 (3)
O2—Cu1—Cu2—O3	−88.77 (9)	C1—C6—C7—O1	−78.2 (3)
O2W—Cu1—Cu2—O3	5.3 (2)	C5—C6—C7—O2	−77.5 (3)
O7—Cu1—Cu2—O8	3.06 (9)	C1—C6—C7—O2	103.9 (3)
O4—Cu1—Cu2—O8	−178.55 (11)	C13—C8—C9—C10	−1.4 (4)
O5—Cu1—Cu2—O8	−86.72 (9)	C8—C9—C10—C11	2.0 (4)
O2—Cu1—Cu2—O8	91.96 (9)	C8—C9—C10—N2	−176.0 (2)
O2W—Cu1—Cu2—O8	−174.0 (2)	O12—N2—C10—C9	−164.6 (3)
O7—Cu1—Cu2—O1	−88.41 (8)	O11—N2—C10—C9	15.0 (4)
O4—Cu1—Cu2—O1	89.98 (9)	O12—N2—C10—C11	17.3 (4)
O5—Cu1—Cu2—O1	−178.20 (11)	O11—N2—C10—C11	−163.1 (2)
O2—Cu1—Cu2—O1	0.49 (8)	C9—C10—C11—C12	−0.4 (4)
O2W—Cu1—Cu2—O1	94.5 (2)	N2—C10—C11—C12	177.5 (2)
O7—Cu1—Cu2—O6	91.96 (8)	C10—C11—C12—C13	−1.7 (4)
O4—Cu1—Cu2—O6	−89.64 (9)	C10—C11—C12—Cl2	−179.3 (2)
O5—Cu1—Cu2—O6	2.18 (9)	C9—C8—C13—C12	−0.7 (4)
O2—Cu1—Cu2—O6	−179.14 (10)	C9—C8—C13—C14	179.7 (3)
O2W—Cu1—Cu2—O6	−85.1 (2)	C11—C12—C13—C8	2.2 (4)
O7—Cu1—Cu2—O1W	−4.3 (3)	Cl2—C12—C13—C8	179.7 (2)
O4—Cu1—Cu2—O1W	174.1 (3)	C11—C12—C13—C14	−178.1 (2)
O5—Cu1—Cu2—O1W	−94.1 (3)	Cl2—C12—C13—C14	−0.7 (4)
O2—Cu1—Cu2—O1W	84.6 (3)	Cu1—O4—C14—O3	−0.8 (4)
O2W—Cu1—Cu2—O1W	178.6 (4)	Cu1—O4—C14—C13	−177.66 (18)
O3—Cu2—O1—C7	84.2 (2)	Cu2—O3—C14—O4	1.8 (4)
O8—Cu2—O1—C7	−84.4 (2)	Cu2—O3—C14—C13	178.69 (17)
O6—Cu2—O1—C7	0.0 (6)	C8—C13—C14—O4	140.1 (3)
O1W—Cu2—O1—C7	−168.7 (2)	C12—C13—C14—O4	−39.6 (4)
Cu1—Cu2—O1—C7	−1.8 (2)	C8—C13—C14—O3	−37.0 (4)
O7—Cu1—O2—C7	86.5 (2)	C12—C13—C14—O3	143.3 (3)
O4—Cu1—O2—C7	−81.7 (2)	C20—C15—C16—C17	−0.2 (5)
O5—Cu1—O2—C7	7.1 (6)	Cl3A—C15—C16—C17	−177.9 (2)
O2W—Cu1—O2—C7	−165.3 (2)	C15—C16—C17—C18	0.4 (5)
Cu2—Cu1—O2—C7	0.6 (2)	C15—C16—C17—N3	−177.6 (3)
O8—Cu2—O3—C14	2.2 (6)	O14—N3—C17—C16	172.8 (3)
O1—Cu2—O3—C14	−85.0 (2)	O13—N3—C17—C16	−5.9 (4)
O6—Cu2—O3—C14	83.6 (2)	O14—N3—C17—C18	−5.3 (4)
O1W—Cu2—O3—C14	−179.9 (2)	O13—N3—C17—C18	175.9 (3)
Cu1—Cu2—O3—C14	−1.5 (2)	C16—C17—C18—C19	−0.5 (4)
O7—Cu1—O4—C14	7.4 (6)	N3—C17—C18—C19	177.6 (2)
O5—Cu1—O4—C14	−83.3 (2)	C17—C18—C19—C20	0.3 (4)
O2—Cu1—O4—C14	84.9 (2)	C17—C18—C19—Cl3B	179.0 (2)
O2W—Cu1—O4—C14	−179.1 (2)	C18—C19—C20—C15	−0.1 (4)
Cu2—Cu1—O4—C14	−0.2 (2)	Cl3B—C19—C20—C15	−178.9 (2)
O7—Cu1—O5—C21	−87.4 (2)	C18—C19—C20—C21	178.5 (3)
O4—Cu1—O5—C21	80.5 (2)	Cl3B—C19—C20—C21	−0.3 (4)
O2—Cu1—O5—C21	−8.0 (6)	C16—C15—C20—C19	0.0 (5)
O2W—Cu1—O5—C21	164.3 (2)	Cl3A—C15—C20—C19	177.8 (2)
Cu2—Cu1—O5—C21	−1.6 (2)	C16—C15—C20—C21	−178.5 (3)
O3—Cu2—O6—C21	−89.5 (2)	Cl3A—C15—C20—C21	−0.8 (4)
O8—Cu2—O6—C21	79.3 (2)	Cu1—O5—C21—O6	−0.7 (4)
O1—Cu2—O6—C21	−5.4 (6)	Cu1—O5—C21—C20	177.50 (19)
O1W—Cu2—O6—C21	163.3 (2)	Cu2—O6—C21—O5	3.7 (4)
Cu1—Cu2—O6—C21	−3.6 (2)	Cu2—O6—C21—C20	−174.52 (18)
O4—Cu1—O7—C28	−10.3 (5)	C19—C20—C21—O5	−99.2 (3)
O5—Cu1—O7—C28	80.7 (2)	C15—C20—C21—O5	79.4 (4)
O2—Cu1—O7—C28	−87.7 (2)	C19—C20—C21—O6	79.2 (4)
O2W—Cu1—O7—C28	176.62 (19)	C15—C20—C21—O6	−102.2 (3)
Cu2—Cu1—O7—C28	−2.63 (19)	C27—C22—C23—C24	1.7 (4)
O3—Cu2—O8—C28	−8.3 (6)	C22—C23—C24—C25	−1.6 (4)
O1—Cu2—O8—C28	78.7 (2)	C22—C23—C24—N4	177.4 (2)
O6—Cu2—O8—C28	−89.8 (2)	O15—N4—C24—C25	159.1 (3)
O1W—Cu2—O8—C28	173.7 (2)	O16—N4—C24—C25	−20.0 (4)
Cu1—Cu2—O8—C28	−4.6 (2)	O15—N4—C24—C23	−20.0 (4)
C6—C1—C2—C3	0.7 (4)	O16—N4—C24—C23	160.9 (3)
C1—C2—C3—C4	−0.3 (4)	C23—C24—C25—C26	0.3 (4)
C1—C2—C3—N1	177.6 (3)	N4—C24—C25—C26	−178.6 (2)
O10—N1—C3—C4	5.1 (4)	C24—C25—C26—C27	0.9 (4)
O9—N1—C3—C4	−176.1 (3)	C24—C25—C26—Cl4	178.0 (2)
O10—N1—C3—C2	−172.9 (3)	C25—C26—C27—C22	−0.8 (4)
O9—N1—C3—C2	5.9 (4)	Cl4—C26—C27—C22	−177.7 (2)
C2—C3—C4—C5	−0.3 (4)	C25—C26—C27—C28	−179.9 (2)
N1—C3—C4—C5	−178.2 (3)	Cl4—C26—C27—C28	3.2 (4)
C3—C4—C5—C6	0.5 (4)	C23—C22—C27—C26	−0.6 (4)
C3—C4—C5—Cl1	−179.2 (2)	C23—C22—C27—C28	178.6 (2)
C4—C5—C6—C1	−0.1 (4)	Cu2—O8—C28—O7	4.3 (4)
Cl1—C5—C6—C1	179.6 (2)	Cu2—O8—C28—C27	−176.45 (17)
C4—C5—C6—C7	−178.7 (3)	Cu1—O7—C28—O8	−0.1 (4)
Cl1—C5—C6—C7	1.0 (3)	Cu1—O7—C28—C27	−179.27 (16)
C2—C1—C6—C5	−0.5 (4)	C26—C27—C28—O8	41.5 (4)
C2—C1—C6—C7	178.1 (3)	C22—C27—C28—O8	−137.6 (3)
Cu2—O1—C7—O2	3.1 (4)	C26—C27—C28—O7	−139.2 (3)
Cu2—O1—C7—C6	−174.68 (17)	C22—C27—C28—O7	41.7 (3)

### Hydrogen-bond geometry (Å, °)

**Table:**

*D*—H···*A*	*D*—H	H···*A*	*D*···*A*	*D*—H···*A*
O1W—H1W1···O13^i^	0.85	2.35	2.910 (3)	124
O1W—H2W1···O2^ii^	0.85	1.99	2.838 (3)	175
O2W—H1W2···O9^iii^	0.82	2.15	2.927 (3)	158
O2W—H2W2···O6^iv^	0.85	1.98	2.826 (3)	173
C1—H1A···Cl1^v^	0.93	2.78	3.417 (3)	127
C4—H4A···O14^vi^	0.93	2.51	3.331 (4)	147
C8—H8A···O12^v^	0.93	2.38	3.269 (4)	159
C18—H18A···O10^vii^	0.93	2.55	3.364 (4)	147
C22—H22A···O16^viii^	0.93	2.36	3.240 (4)	158
C23—H23A···O2W^i^	0.93	2.51	3.385 (3)	157

Symmetry codes: (i) *x*, −*y*+2, *z*−1/2; (ii) *x*+1, *y*, *z*; (iii) *x*, −*y*+1, *z*+1/2; (iv) *x*−1, *y*, *z*; (v) *x*, −*y*+1, *z*−1/2; (vi) *x*−1, *y*−1, *z*; (vii) *x*+1, *y*+1, *z*; (viii) *x*, −*y*+2, *z*+1/2.
